# Combining evidence for and against pathogenicity for variants in cancer susceptibility genes: CanVIG-UK consensus recommendations

**DOI:** 10.1136/jmedgenet-2020-107248

**Published:** 2020-11-18

**Authors:** Alice Garrett, Miranda Durkie, Alison Callaway, George J Burghel, Rachel Robinson, James Drummond, Bethany Torr, Cankut Cubuk, Ian R Berry, Andrew J Wallace, Sian Ellard, Diana M Eccles, Marc Tischkowitz, Helen Hanson, Clare Turnbull, CanVIG-UK, Stephen Abbs

**Affiliations:** 1 Division of Genetics and Epidemiology, Institute of Cancer Research, Sutton, London, UK; 2 Sheffield Diagnostic Genetics Service, Sheffield Children's NHS Foundation Trust, Sheffield, UK; 3 Wessex Regional Genetics Laboratory, Salisbury Hospital NHS Foundation Trust, Salisbury, Wiltshire, UK; 4 Human Genetics and Genomic Medicine, University of Southampton Faculty of Medicine, Southampton, UK; 5 Manchester Centre for Genomic Medicine and NW Laboratory Genetics Hub, Manchester University NHS Foundation Trust, Manchester, UK; 6 Yorkshire Regional Genetics Service, Leeds Teaching Hospitals NHS Trust, Leeds, UK; 7 East Anglian Medical Genetics Service, Addenbrooke’s Hospital, Cambridge, Cambridgeshire, UK; 8 Department of Molecular Genetics, Royal Devon and Exeter NHS Foundation Trust, Exeter, Devon, UK; 9 Cancer Sciences Research Group, University of Southampton Faculty of Medicine, Southampton, UK; 10 Department of Medical Genetics and National Institute for Health Research Cambridge Biomedical Research Centre, University of Cambridge, Cambridge, Cambridgeshire, UK; 11 Department of Clinical Genetics, St George's University Hospitals NHS Foundation Trust, London, UK; 12 Cancer Genetics Unit, Royal Marsden NHS Foundation Trust, London, UK

**Keywords:** genetics, genomics, genetic testing, genetics, medical, genetic variation

## Abstract

Accurate classification of variants in cancer susceptibility genes (CSGs) is key for correct estimation of cancer risk and management of patients. Consistency in the weighting assigned to individual elements of evidence has been much improved by the American College of Medical Genetics (ACMG) 2015 framework for variant classification, UK Association for Clinical Genomic Science (UK-ACGS) Best Practice Guidelines and subsequent Cancer Variant Interpretation Group UK (CanVIG-UK) consensus specification for CSGs. However, considerable inconsistency persists regarding practice in the combination of evidence elements. CanVIG-UK is a national subspecialist multidisciplinary network for cancer susceptibility genomic variant interpretation, comprising clinical scientist and clinical geneticist representation from each of the 25 diagnostic laboratories/clinical genetic units across the UK and Republic of Ireland. Here, we summarise the aggregated evidence elements and combinations possible within different variant classification schemata currently employed for CSGs (ACMG, UK-ACGS, CanVIG-UK and ClinGen gene-specific guidance for PTEN, TP53 and CDH1). We present consensus recommendations from CanVIG-UK regarding (1) consistent scoring for combinations of evidence elements using a validated numerical ‘exponent score’ (2) new combinations of evidence elements constituting likely pathogenic’ and ‘pathogenic’ classification categories, (3) which evidence elements can and cannot be used in combination for specific variant types and (4) classification of variants for which there are evidence elements for both pathogenicity and benignity.

## Background

### Variant interpretation in cancer susceptibility genetics

Accurate classification of variants in cancer susceptibility genes (CSGs) is key for the correct estimation of cancer risk and management of patients with potential cancer predisposition.^1^ There are specific interventions that would only be offered if a patient were at very substantially increased risk of cancer; most typically, only those carrying a (likely) pathogenic variant in a relevant CSG. Incorrect classification of a variant as (likely) pathogenic can thus lead to ‘overmanagement’, for example, the inappropriate performance of risk-reducing surgery. Conversely, there are significant potential sequelae of underclassification of a pathogenic variant as a ‘variant of uncertain significance’ (VUS). For example, for a variant in BRCA1/BRCA2, first, the patient with cancer may not be eligible for cancer treatments from which they would likely benefit, for example, platinum-based chemotherapy and/or poly ADP ribose polymerase (PARP) inhibitors. Second, the patient with cancer may not be eligible for risk-reduction interventions appropriate to their true level of risk, for example, contralateral risk-reducing mastectomy or salpingo-oopherectomy. Third, family members will be unable to access presymptomatic testing by which their cancer risk can be clarified as either near-population or substantially elevated.^2^ Furthermore, the results of CSG analysis may be used for prenatal testing or preimplantation genetic diagnosis, for which accuracy of genetic test interpretation is also crucial. This difficult balance between reducing categorisation as uncertain and avoiding ‘false positives’ is a challenging tightrope in clinical cancer genetics, as indeed in other areas of genetics and medicine more widely.

### 2015 American College of Medical Genetics (ACMG) variant interpretation framework

A variety of evidence types can contribute to assertions of pathogenicity or benignity, for example, the number of independent cases with a characteristic phenotype, familial segregation data, frequency in population controls and functional analyses. Historically, appropriation of disparate evidence elements could differ widely between diagnostic laboratories and produce discrepant classifications. To advance consistency in diagnostic variant interpretation, in 2015, the ACMG published a framework for variant classification.^3^ In 2016, it was agreed by the UK Association for Clinical Genomic Science (UK-ACGS) to adopt formally across NHS molecular diagnostics the ACMG framework for variant interpretation. Each year, a detailed UK ACMG-based specification is published, ‘The UK-ACGS Best Practice Guidelines for Variant Classification in Rare Disease’.^4^

### Cancer Variant Interpretation Group UK (CanVIG-UK)

CanVIG-UK was established in 2017 as part of the UK-ACGS activity supporting adoption and dissemination of the ACMG framework for variant interpretation.^5^ CanVIG-UK currently comprises >100 clinical scientists, clinical geneticists and genetic counsellors, with representation from each of the 25 Molecular Diagnostic Laboratories and Clinical Genetics Services of the UK (NHS) and Republic of Ireland. The group meets monthly to undertake multidisciplinary review and interpretation of problematic clinically detected variants. CanVIG-UK maintains an annually updated consensus specification for CSGs of the UK-ACGS Best Practice Guidelines for Variant Classification in Rare Disease (hereafter termed the CanVIG-UK specification).^5^

### Combining evidence items under the ACMG framework

The ACMG framework has greatly improved the consistency with which variants in CSGs are classified within the UK molecular genetics laboratory community. Nevertheless, within the framework, several areas are ambiguous, undefined or make reference to the need for ‘expert judgment’.^3^ Additional evidence elements not present in the original 2015 ACMG framework have been introduced in recent specifications of the framework by ClinGen expert groups, as well as the UK-ACGS and CanVIG-UK.^4–9^

Frequently encountered within CanVIG-UK are variants for which there is uncertainty and inconsistency regarding the combination of multiple evidence elements, in particular those for which (1) the evidence elements available do not conform to any of the combinations specified in the original ACMG framework; (2) a proposed combination of evidence elements is of contentious legitimacy; and (3) there are conflicting evidence elements, that is, towards both pathogenicity and benignity.

We thus sought within CanVIG-UK to improve consistency in combination of evidence elements within the ACMG framework, addressing the following five objectives:

- To establish an objective numerical system for combining evidence elements.
- To evaluate the maximum number of evidence items attainable via new ACMG-based specifications.
- To define specifically permitted and non-permitted combinations of evidence elements.
- To define all new combinations of evidence elements attainable using new ACMG-based specifications.
- To establish consistent practice in classification of variants with discordant (conflicting) evidence elements.

## Objective 1: to establish an objective numerical system for combining evidence elements within the ACMG framework

### Background and approach

In the original 2015 ACMG framework, four evidence strength levels were defined: supporting (P), moderate (M), strong (S) and very strong (VS).^3^ Empirical combinations of evidence elements were set out for classification categories of pathogenic (eight combinations), likely pathogenic (six combinations), likely benign (two combinations) and benign (two combinations).

Although developed empirically through clinical consensus, the ACMG framework was subsequently demonstrated by Tavtigian *et al* to conform well to a Bayesian structure, namely, that prior probability × likelihood ratio = posterior probability, where the evidence strength levels were related in an exponential series to a base of 2.08.^10^ We sought to evolve the Tavtigian Bayesian metastructure into a numerical ‘scoring’ system to provide for clinical users an easy-to-use system for the combining of evidence elements that is consistent and objective.

### Outcome

Derived from the Tavtigian Bayesian metastructure and consistent with the UK-ACGS Best Practice Guidelines for Variant Classification in Rare Disease 2020, we defined an ‘exponent scoring system’, similar to the approach taken by Tavtigian *et al* in a recent adaptation of their original metastructure.^4 10 11^ Evidence elements were allocated points according to evidence strength level: (towards pathogenicity) VS (eight points), S (four points), M (two points), and P (one point) and (towards benignity) S (−4 points) and P (−1 points) (table 1A). Arithmetic summing of the exponent scores for the contributing evidence elements generates an ‘exponent sum’. Assignment of a particular classification is predicated on attainment of an exponent sum threshold value of 6–9 (likely pathogenic), ≥10 (pathogenic), (−1)−(−5) (likely benign) and ≤−6 (benign) (table 1B).

**Table 1:** Probability calculations for attaining (A) evidence elements and (B) classification categories

(A)			
Evidence strength	Odds of pathogenicity		Exponent score
Very Strong (VS)	350	2.08^8^	8
Strong (S)	18.7	2.08^4^	4
Moderate (M)	4.33	2.08^2^	2
Supporting (P)	2.08	2.08^1^	1

(B)				
Classification category	Prior probability	Combined odds of pathogenicity	Exponent sum	Posterior probability
Pathogenic	0.1	1514 (2.08^+10^)	≥10	0.99
Likely pathogenic		81 (2.08^+6^)	6–9	0.90
Likely benign		0.48 (2.08^−1^)	(−1)−(−5)	0.05
Benign		0.01 (2.08^−6^)	≤−6	0.001*

*The posterior probability attained with an exponent sum of −6 has been rounded down to 0.001, consistent with the UK Association for Clinical Genomic Science Best Practice Guidelines for Variant Classification in Rare Disease 2020.^4^

### Discussion

Conversion of the tally-based 2015 ACMG framework into a numerical exponent scoring system (derived from the Tavtigian Bayesian metastructure) is a useful evolution of the ACMG framework allowing rapid calculation of variant classification category. It is designed to augment, not replace the five-level classification system. In addition, the exponent scoring system:

- Enables delineation of previously undescribed legitimate combinations of evidence elements of equivalent numerical posterior probability.
- Highlights the incongruity in the numerical posterior probability for some combinations of evidence elements in the original 2015 ACMG framework, as previously described by Tavtigian *et al.* 10
- Allows objective stratification of the evidence strength for likely pathogenic variants (exponent score sum range 6–9). The exponent system provides a clearer and more consistent numerical language with which to continue dialogue regarding management quandaries about likely pathogenic variants being of ‘lower confidence’ and ‘higher confidence’. This typically comprises contexts in which a higher bar of evidence is argued to be desirable (eg, prenatal testing).
- Likewise allows objective stratification of evidence strength for ‘uncertain’ variants (exponent score sum range 0–5) (table 2, as described in the UK-ACGS Best Practice Guidelines for Variant Classification in Rare Disease 2020).^4^ Again, the exponent system provides a clearer and more consistent numerical language with which to debate longstanding clinical quandaries/scenarios, such as:
  - Quantifying the magnitude of additional evidence required for a VUS to achieve a classification of likely pathogenic (eg, ‘a four-point-VUS, for which two more exponent points are required for upclassification to likely pathogenic’).
  - Defining the swathe of VUSs for which ‘active’ variant monitoring is indicated (eg, perhaps all five-point VUSs).
  - Differential evidence requirements in oncology between the therapeutic context and the context of familial risk prediction. It has been argued in oncology that evidence of therapeutic actionability for some somatic biomarkers in use may be significantly weaker than for others, for example, atypical BRAF variants as indication for vemurafenib administration or an Allred score of 4 as indication for tamoxifen administration.^12–14^ Whilst it has been argued that germline variants of posterior probability of pathogenicity of 0.8-0.9 (80%–90%) could be equivalently eligible as therapeutic biomarkers (while not used for genetic risk prediction), any changes to current practice would require very careful consideration of potential clinical pitfalls and detailed economic evaluation.

**Table 2:** Five strengths of variant of uncertain significance, annotated by potential evidence combinations, posterior probability of pathogenicity, ‘exponent sum’ and UK-ACGS Best Practice Guidelines for Variant Classification in Rare Disease descriptor^4^

Exponent score for evidence elements	Exponent sum	Overall (posterior) probability of pathogenicity (%)	UK-ACGS descriptor
Towards pathogenicity: 8 (very strong), 4 (strong), 2 (moderate), 1 (supporting) towards benignity: −4 (strong), −1 (supporting)			
4+1	5	0.812 (81.2)	Hot VUS
2+2+1			
2+1+1+1			
1+1+1+1+1			
4	4	0.675 (67.5)	Warm VUS
2+2			
2+1+1			
1+1+1+1			
2+1	3	0.500 (50.0)	Tepid VUS
1+1+1			
2	2	0.325 (32.5)	Cool VUS
1+1			
1	1	0.188 (18.8)	Ice-cold VUS

UK-ACGS, UK Association for Clinical Genomic Science; VUS, variant of uncertain significance.

Key to implementation of the Bayesian metastructure (and indeed the 2015 ACMG framework) is recognition that the overall (posterior) probability of pathogenicity is dependent not just on the aggregate likelihood ratio of the contributory evidence elements but also on the prior probability of pathogenicity. The exponent sums presented correspond to the respective posterior probability thresholds only in the context of a specified prior probability of 0.1 (10%). This approximates to clinical analysis of a limited gene set in a proband with a suspected Mendelian disorder.^10 15^ In the context of a substantially lower prior probability (absence of phenotype and/or examination of a much larger gene set), additional evidence elements will be required to attain a specified posterior probability.^16 17^ Potential methods for estimating the prior probability of pathogenicity in different contexts are under development by several groups; integration into clinical variant interpretation frameworks is a priority for future work.

### CanVIG-UK consensus recommendations

- The exponent scoring system (derived from the Tavtigian Bayesian metastructure) is a legitimate means of summing evidence elements from the 2015 ACMG framework.
- Where there is a prior probability of ~0.1 (10%), an exponent sum of ≥6 equates to a classification class of likely pathogenic (ie, >0.9 (90%) posterior probability of pathogenicity), ≥10 of pathogenic (>0.99 (99%) posterior probability of pathogenicity), ≤−1 of likely benign (<0.1 (10%) posterior probability of pathogenicity) and ≤−6 of benign (<0.001 (0.01%) posterior probability of pathogenicity).
- A higher exponent sum is required to attain the equivalent posterior probabilities of pathogenicity where the prior probability of an underlying Mendelian mechanism is significantly less than 0.1 (10%) eg, variants identified as ‘additional findings’ on sequencing for an alternative indication.
- Five-point (hot) VUSs in well-studied CSGs (BRCA1/BRCA2 and mismatch repair (MMR) genes) should be discussed by a multidisciplinary team for potential inclusion on clinical reports. There are rapid-paced international research endeavours relating to these genes through which upclassification is quite possible.^4 18^

## Objective 2: to evaluate the maximum number of evidence items attainable via new ACMG-based specifications

### Background and approach

In the 2015 ACMG framework, 30 different evidence elements were specified (including three different evidence strength levels for PP1).^3^ However, evidence elements have been applied at additional and/or different strength levels in subsequent ACMG-based specifications. Thus, we reviewed the range and total number of evidence elements now attainable via the newer ACMG-based specifications currently used by CanVIG-UK for the interpretation of variants in CSGs (table 3 and online supplemental table 1).

10.1136/jmedgenet-2020-107248.supp1 Supplementary data

**Table 3:** Total evidence elements included within current versions of ACMG-based specifications used by CanVIG-UK for CSG variant interpretation

Specification	Evidence for pathogenicity				Evidence for benignity			Total
	Very strong	Strong	Moderate	Supporting	Supporting	Strong	Stand-alone	
ACMG framework 2015^3^	1	5	7	5	7	4	1	30
UK-ACGS rare disease specification 2020^4^	4	10	13	15	7	4	1	54
CanVIG-UK specification 2020^5^	5	9	13	15	9	5	1	57
ClinGen CDH1 specification V2^8^	4	7	7	5	5	4	2	34
ClinGen PTEN specification V2^7^	4	6	7	5	8	4	1	35
ClinGen TP53 specification V1^6^	3	7	10	7	5	4	1	37

ACMG, American College of Medical Genetics; CanVIG-UK, Cancer Variant Interpretation Group UK; CSG, cancer susceptibility gene; UK-ACGS, UK Association for Clinical Genomic Science.

### Outcome

#### Discussion

For each of the ACMG-based specifications used for CSG variant interpretation, the number of potential evidence elements at each strength level was increased compared with the original 2015 ACMG framework. Accordingly, a greater number of possible combinations of evidence elements will be attainable than was possible using the original 2015 ACMG framework.

## Objective 3: to define which specific combinations of evidence elements are permitted

### Background and approach

Within the 2015 ACMG framework and subsequent ACMG-based specifications, several of the evidence elements are (1) non-independent of each other and/or (2) incompatible with regard to the specific variant types to which they can be applied. Accordingly, the combinations of evidence elements actually attainable in practice are restricted, and there is inconsistency regarding which evidence elements are used together. We identified pairs of evidence elements for which the legitimacy of combination is debatable and established, within CanVIG-UK, consensus on the legitimacy of combination (table 4). We then applied these restrictions to calculate the final number of combinable evidence elements per variant type as described by Brnich *et al* 19 (table 5).

**Table 4:** Permissible and non-permissible combinations of concordant evidence elements (CanVIG-UK consensus)

Theme	Evidence elements	CanVIG consensus	Notes, references
In silico + functional data	PS3 (functional) and PP3 (in silico)	✔ **X**	Co-usage permitted for assays of protein function Co-usage not permitted for assays of splicing; in silico evidence incorporated into PS3 for assays of splicing (as per ACGS guidance)^4 5 19 21 22^
	BS3 (functional) and BP4 (in silico)	✔ **X**	
	PM1 (hot spot) and PP3 (in silico)	✔	Co-usage permitted; regional enrichment and in silico prediction largely different evidence types (and evidence from three tools generally incorporated)^15^
In silico + variant mechanism	BP4 (in silico) and BP7 (silent variant)	✔	Co-usage permitted for synonymous and intronic variants (splicing effect must be excluded)
	PVS1 (null variant) and PP3 (in silico)	**X**	Co-usage not permitted; in silico predictors often incorporate variant mechanism. The strongest legitimate evidence item should be selected for inclusion.^23^
	PM4 (protein length changes) and PP3 (in silico)	**X**	
	BP3 (in-frame del/ins) & BP4 (in silico)	**X**	
Use of population data	PS4 (case control) and PM2 (absence in controls)	✔	Co-usage permitted, provided that *a different* source of population data is used for each (this can comprise two *predefined* partitions of gnomAD)^5^
Phenotypic specificity	PS4 (case control) and PP4 (phenotype specificity)	✔	Co-usage permitted, provided that there is a schema predefining distinct data types used for each, thus preventing ‘double counting’ of phenotypic features (eg PP4 is applied for molecular/tumour assay data, indicating specificity at gene level); PS4 is applied at patient level, counting the strength of features in cases and the number of cases/families^5^
Scaled evidence items	PS2 (de novo confirmed) and PM6 (de novo not confirmed)	**X**	Co-usage not permitted; either PS2 or PM6 is used, depending on aggregate observations of de novo occurrence (ClinGen Sequence Variant Interpretation Working Group)^24^
Overlapping/ related evidence	PS1 (same amino acid) and PM5 (same residue)	**X**	Co-usage not permitted; the strongest legitimate evidence item should be selected for inclusion
	PM1 (hot spot) and PP2 (low missense rate)	✔	Co-usage permitted; there may be both a low rate of benign missense variation at a whole gene/gene region level and a specific mutational hot spot/functional domain
	PVS1 (null variant) and PM4 (protein length changes)	**X**	Co-usage not permitted; this is double counting of two end points of deleterious effect^23^
	PVS1 (null variant) and PS3 (functional)	✔ **X**	Co-usage not permitted for canonical splice variants or non-canonical splice variants (experimental evidence cannot be scored using PS3 if PVS1 is used)^23^ Co-usage is permitted for assays of protein function where the evidence is not double-counted (eg, truncating variant in last exon of gene scored as PVS1_mod can be combined with PS3 for experimental evidence, demonstrating a significant effect on protein function)
	PS1 (same amino acid) and PM1 (hot spot)	✔*	Co-usage permitted; definition of a hot spot is predicated on multiple well-documented pre-existing pathogenic variants. This may include those at the same amino acid
	PM5 (same residue) and PM1 (hot spot)	✔*	
Alternative explanation for phenotype	BP2 (observed with pathogenic variant) and BP5 (alternative cause)	**X**	Co-usage not permitted; These evidence elements both pertain to presence of an alternative genetic cause (BP2 in the same gene and BP5 in a different gene)

*PM1 should therefore be used at supporting level if used in combination with PS1 or PM5.^15^

ACGS, Association for Clinical Genomic Science; CanVIG-UK, Cancer Variant Interpretation Group UK.

**Table 5:** Maximum number of evidence elements attainable by variant type using the CanVIG-UK specification

Variant type	Maximum number of pieces of evidence at each strength						
	Pathogenicity				Benignity		
	Very strong	Strong	Moderate	Supporting	Supporting	Strong	Standalone
Truncating	4	7	8	9	4	5	1
Missense	3	7	9	12	6	5	1
In-frame indel	3	6	9	10	5	5	1
Synonymous	3	6	7	9	6	5	1

PP5 and BP6 (reputable source) are permitted by the CanVIG-UK specification 2020 at supporting level and so are included in the totals above, despite not being permitted by Brnich *et al* 19

CanVIG-UK, Cancer Variant Interpretation Group UK.

### Outcome

#### Discussion

Gene-specific ACMG-based specifications vary in the combinations of evidence items they permit. It is unclear to what extent this variation reflects between-gene clinical–biological heterogeneity versus differences in approaches between expert groups.

### CanVIG-UK consensus recommendation

Pairs of evidence elements should be combined as per table 4. This will reduce overcounting of non-orthogonal evidence items derived from a common underlying source/phenomenon, thus improving the validity of evidence combination and consistency of classifications between classifiers.

## Objective 4: to define all new combinations of evidence elements attainable using new ACMG-based specifications

### Background and approach

In the 2015 ACMG framework, eight evidence combinations were provided for pathogenic, six for likely pathogenic, two for likely benign and two for benign. Using our updated counts of total possible numbers of evidence elements (table 6), using the exponent scoring system (derived from the Tavtigian Bayesian metastructure), we sought to identify whether additional combinations of numerically equivalent evidence might be possible.

**Table 6:** New and existing combinations of exponent scores potentially leading to overall CanVIG-UK variant classifications

2015 ACMG framework assignation of variant class	Exponent score of evidence elements	Exponent sum	CanVIG-UK assignation of variant class
	Towards pathogenicity: 8 (very strong), 4 (strong), 2 (moderate), 1 (supporting); towards benignity: −4 (strong), −1 (supporting)		
Pathogenic (ia)	8+4	12	Pathogenic (ia)
Pathogenic (ib)	8+2+2	12	Pathogenic (ib)
Pathogenic (ic)	8+2+1	11	Pathogenic (ic)
Pathogenic (id)	8+1+1	10	Pathogenic (id)
L/pathogenic (i)	8+2	10	Pathogenic (ie)
Pathogenic (iiia)	4+2+2+2	10	Pathogenic (iia)
Pathogenic (iiib)	4+2+2+1+1	10	Pathogenic (iib)
Pathogenic (iiic)	4+2+1+1+1+1	10	Pathogenic (iic)
Not included	4+4+2	10	Pathogenic (iiia)
Not included	4+4+1+1	10	Pathogenic (iiib)
Not included	2+2+2+2+2	10	Pathogenic (iv)
Not included	2+2+2+2+1+1	10	Pathogenic (v)
Not included	2+2+2+1+1+1+1	10	Pathogenic (vi)
Not included	2+2+1+1+1+1+1+1	10	Pathogenic (vii)
Not included	2+1+1+1+1+1+1+1+1	10	Pathogenic (viii)
Not included	1+1+1+1+1+1+1+1+1+1	10	Pathogenic (viii)
Pathogenic (ii)	4+4	8	L/pathogenic (v)
L/pathogenic (ii)	4+2	6	L/pathogenic (i)
L/pathogenic (iii)	4+1+1	6	L/pathogenic (ii)
L/pathogenic (iv)	2+2+2	6	L/pathogenic (iii)
L/pathogenic (v)	2+2+1+1	6	L/pathogenic (iv)
L/pathogenic (vi)	2+1+1+1+1	6	L/pathogenic (vi)
Not included	8+1	9	L/pathogenic (vii)
Not included	8	8	N/A: single evidence element
Not included	1+1+1+1+1+1	6	L/pathogenic (viii)
L/benign (i)	(−1)+(−4)	-5	L/benign (i)
L/benign (ii)	(−1)+(−1)	-2	L/benign (ii)
Not included	(−1)	-1	N/A: single evidence element
Not included	(−4)	-4	N/A: single evidence element
Benign (i)	(−4)+(−4)	-8	Benign (i)

Classifications in dark grey are those present in the 2015 ACMG framework that are inconsistent with the Tavtigian Bayesian metastructure, and those in light grey are new combinations generated using the exponent scoring system.

ACGS, Association for Clinical Genomic Science; ACMG, American College of Medical Genetics; CanVIG-UK, Cancer Variant Interpretation Group UK; L, likely; N/A, not applicable.

### Outcome

As previously described by Tavtigian *et al* and the UK-ACGS Best Practice Guidelines for Variant Classification in Rare Disease 2020, we confirmed that two combinations in the 2015 ACMG framework were incongruous with the exponent scoring system, (1) one ascribed as pathogenic for which the exponent score is only eight and (2) one ascribed as likely pathogenic for which the exponent score is 10.^4 10^ Consistent with restrictions in combination due to non-permissibility (table 4) and/or incompatible variant types (table 5), we confirmed validity of nine new combinations for pathogenic and three for likely pathogenic.

### Discussion

It was the overall consensus of the CanVIG-UK group to retain the requirement for at least two items of evidence to provide buffer against false-positive classification of a variant as likely benign/likely pathogenic via a spurious evidence item. Thus, although a sufficient exponent sum is attained with a single evidence item, classification out of VUS is not permitted in these instances: (1) likely pathogenic with a single very strong evidence element, (2) likely benign with a single supporting/strong evidence element.

### CanVIG-UK consensus recommendations

- The exponent sum threshold for a classification category can be attained via any combination of two or more concordant evidence elements.
- Variants should not be classified as pathogenic, likely pathogenic, benign or likely benign on the basis of a single evidence item, except BA1 (‘stand-alone evidence’ for benignity).^20^

## Objective 5: to establish consistent practice in classification of variants with discordant (conflicting) evidence elements

### Background and approach

In the original 2015 ACMG framework, the recommendation was to classify as uncertain any variant for which there was discordancy in the evidence elements.^3^ We sought through application of the exponent scoring system to undertake more direct numerical evaluation of scenarios of discordancy. We considered three potential approaches towards classification of variants with discordant evidence: (1) we could classify all variants with discordant evidence as VUSs, as per the original ACMG framework; (2) we could use agnostically the net exponent score generated from combination of evidence elements as laid out in the original Tavtigian *et al* paper, regardless of the extent of conflicting evidence; (3) we could use the net exponent score but with rules-based restriction regarding the maximum number of evidence elements ‘discordant’ with the final classification.^10^

### Outcome

These three approaches were reviewed within CanVIG-UK through application to a number of exemplar variants. Consensus opinion was for option 3 as laid out in the following consensus recommendations and in table 7.

**Table 7:** Examples of potential combinations of conflicting evidence and the resultant CanVIG-UK classification that would be given

Evidence elements for pathogenicity	Evidence elements for benignity	Net exponent score	CanVIG-UK assignation of variant class
Towards pathogenicity: 8 (very strong), 4 (strong), 2 (moderate), 1 (supporting); towards benignity: −4 (strong), −1 (supporting)			
8+4	(−1)	11	Likely pathogenic
8+4	(−1)+(−1)	10	VUS
8+4	(−4)	8	VUS
4+4	(−1)	7	Likely pathogenic
4+4+1	(−1)+(−1)	7	VUS
1	(−4)	-3	Likely benign
1+1	(−4)+(−4)	-6	VUS

CanVIG-UK, Cancer Variant Interpretation Group UK; VUS, Variant of Uncertain Significance.

### Discussion

In variant classification, we seek to balance the clinical benefit of classification of a variant out of the ‘VUS’ category against the harms of erroneous misclassification. Variants classified as likely pathogenic have a 90%–99% likelihood of pathogenicity: this group should thus contain a ~5% (1%–10%) frequency of variants that are truly benign and have been misclassified as likely pathogenic. Variants classified as likely benign have a 0.1%–10% likelihood of truly being pathogenic. Thus, while downclassification of variants in CSGs will and should occur periodically in practice, it is important to recognise the consequent clinical disruption, particularly if multiple unaffected family members have undertaken risk-reducing surgery.

There would be a clear rationale for agnostic use of the net exponent score if all evidence was of unquestionable provenance and guaranteed to be wholly correct. In practice, in clinical observations, laboratory assays and/or published reports, there is always potential for error in evidence generation or communication. The presence of discordant evidence elements will occur by chance through statistical distribution of true results; it may also be an indicator towards error within one of the evidence items. Discordant results between clinical findings and laboratory results may also be an indicator of intermediate penetrance. Overall in CanVIG-UK, we adopted a strategy whereby classification from VUS to another class was permitted, providing the conflicting evidence did not exceed a single supporting evidence element.

PM2 (absence in controls) provides evidence of rarity, rather than evidence against benignity and can thus be ignored when calculating the *net* exponent sum for benignity. Pathogenic variants will necessarily be rare (except occasional founders); the frequency of benign variants will vary from very common to very rare.

### CanVIG-UK consensus recommendations

- Where there is discordant evidence, regardless of the net exponent score, the classification class cannot exceed likely pathogenic or likely benign. Variants with discordant evidence items should not be classified as pathogenic or benign.
- Conflicting evidence items should be combined to calculate a net exponent sum using the ‘exponent score system’ (derived from the Tavtigian Bayesian metastructure). In the presence of discordant evidence, providing there is no more than one discordant evidence element at no more than supporting level, if the net exponent sum is >6, the variant can be assigned the variant class of ‘likely pathogenic’; if the net exponent sum is <−1, the variant can be assigned the variant class of ‘likely benign’.
- PM2 can be ignored when calculating the ***net*** exponent sum for benignity.

## Conclusion

Clinical variant interpretation is a rapidly evolving field. The 2015 ACMG framework has provided an invaluable common framework for which there has been wide international adoption and improved congruity of approach. ClinGen gene-specific ACMG-based specifications in cancer susceptibility are currently finalised for PTEN, TP53 and CDH1.^6–8^ CanVIG-UK is a national sub-specialty multi-disciplinary genomic network through which we have attained consensus and improved consistency within the UK clinical-laboratory community around application of the 2015 ACMG framework for these and other CSGs. We here have presented CanVIG-UK consensus recommendations for combining evidence elements for classification of variants in CSGs including (i) an exponent scoring system for quantitative combination of evidence elements (ii) permissible and non-permissible evidence element combinations (iii) new combinations of evidence elements attaining classification categories of likely pathogenic and pathogenic’ and (iv) rules for combination of discordant (conflicting) evidence elements.
