# Supplementary material for: Combining evidence for and against pathogenicity for variants in cancer susceptibility genes: CanVIG-UK consensus recommendations
Source: J Med Genet. 2020 Nov 18;58(5):297–304. doi: 10.1136/jmedgenet-2020-107248 (PMC8086256; doi:10.1136/jmedgenet-2020-107248)
Supplement: Supplementary data [file jmedgenet-2020-107248supp001.pdf]

	Criteria	Very strong_path	Strong_path	Moderate_path	Supporting_path	Supporting_benign	Strong_benign	Standalone_benign
**Population**	Population Freq (BA1/BS1)					PT	AG AS PT CV TP	ALL*
	Population Freq (PM2)			AG AS CV CD PT	AS CV TP			
	Observation in controls inconsistent with disease penetrance (BS2)					CD PT CV TP	ALL	
	Case control freq (PS4)	CV CD PT	ALL	AS CV CD PT TP	AS CV CD PT TP			
**Computational/predictive**	Multiple lines of computational evidence (BP4 or PP3)			CD TP	ALL	ALL		
	Missense in gene where only truncating cause disease (BP1)					AG AS CV		
	Silent variant with non predicted splice impact (BP7)					ALL		
	In-frame indels in repetitive region without known function (BP3)					AG AS CV		
	Novel missense change at an amino acid residue where a different pathogenic missense change has been seen before (PM5)			AG AS CV PT TP	AS CV TP			
	Protein length changing variant (PM4)			AG AS CD CV PT	AS CV			
	Same amino acid change as an established pathogenic variant (PS1)		ALL	AS CV TP	AS CV			
	Predicted null variant in a gene where LOF is a known mechanism of disease (PVS1)	ALL	AS CV CD TP	AS CV CD TP	AS CV CD TP			

	Missense in gene with low rate of benign missense variants and path. Missenses common (PP2)				AG AS CV PT			
	Well-established functional studies (BS3 or PS3)	CV	ALL	AS CV TP	AS CV CD PT	CV PT TP	ALL	
**Functional**	Mutational hot spot or well-studied functional domain without benign variation (PM1)		AS	AG AS CV PT TP	AS CV			
**Segregation**	Cosegregation with disease in multiple affected family members (BS4 or PP1)		ALL	ALL	ALL	PT	ALL	
	De novo (without paternity & maternity confirmed) (PM6)	AS CD CV PT TP	AS CD CV PT TP	ALL	AS CV TP			
**De novo**	De novo (paternity and maternity confirmed) (PS2)	AS CD CV PT TP	ALL	AS CV TP				
	Observed in trans with a dominant variant OR Observed in cis with a pathogenic variant (BP2)					ALL	CD CV	
**Allelic**	For recessive disorders, detected in trans with a pathogenic variant (PM3)	AS	AS CV	AG AS CV	AS CV			
**Other database**	Reputable source (BP6 or PP5)				AG AS CV	AG AS CV		
	Found in case with an alternate cause (BP5)					AG AS CD CV PT		
**Other**	Highly specific phenotype (PP4)		AS CV	AS CV	AG AS CV			

**Key**	ALL	Present in ACMG, ACGS, CanVIG, CDH1, TP53 and PTEN guidance
	AG	ACMG framework 2015[3]
	AS	UK-ACGS rare disease specification 2020[4]
	CV	CanVIG-UK specification 2020[5]
	CD	ClinGen CDH1 specification V2[8]
	PT	ClinGen PTEN specification V2[7]
	TP	ClinGen TP53 specification V1[6]

*CDH1 guidance recommends both BA1 and BS1 to be used as standalone
